# Evaluation of a rapid quantitative determination method of PSA concentration with gold immunochromatographic strips

**DOI:** 10.1186/s12894-015-0105-7

**Published:** 2015-11-03

**Authors:** Cheng-Ching Wu, Hung-Yu Lin, Chao-Ping Wang, Li-Fen Lu, Teng-Hung Yu, Wei-Chin Hung, Jer-Yiing Houng, Fu-Mei Chung, Yau-Jiunn Lee, Jin-Jia Hu

**Affiliations:** Institute of Biomedical Engineering, National Cheng Kung University, Tainan, 70101 Taiwan; Department of Urology, E-Da Hospital, I-Shou University, Kaohsiung, 82445 Taiwan; Division of Cardiac Surgery, Department of Surgery, E-Da Hospital, I-Shou University, Kaohsiung, 82445 Taiwan; Department of Medical Nutrition, Institute of Biotechnology and Chemical Engineering and I-Shou University, Kaohsiung, 82445 Taiwan; Lee’s Endocrinology Clinic, Pingtung, 90000 Taiwan; Division of Cardiology, Department of Internal Medicine, E-Da Hospital, I-Shou University, Kaohsiung, 82445 Taiwan; School of Medicine for International Students, I-Shou University, Kaohsiung, 82445 Taiwan

**Keywords:** Prostate specific antigen, Prostate cancer, Chromogenic rapid test reader, Gold immunochromatographic strip

## Abstract

**Background:**

Prostate cancer remains the most common cancer in men. Qualitative or semi-quantitative immunochromatographic measurements of prostate specific antigen (PSA) have been shown to be simple, noninvasive and feasible. The aim of this study was to evaluate an optimized gold immunochromatographic strip device for the detection of PSA, in which the results can be analysed using a Chromogenic Rapid Test Reader to quantitatively assess the test results.

**Methods:**

This reader measures the reflectance of the signal line via a charge-coupled device camera. For quantitative analysis, PSA concentration was computed via a calibration equation. Capillary blood samples from 305 men were evaluated, and two independent observers interpreted the test results after 12 min. Blood samples were also collected and tested with a conventional quantitative assay.

**Results:**

Sensitivity, specificity, positive and negative predictive values, and accuracy of the PSA rapid quantitative test system were 100, 96.6, 89.5, 100, and 97.4 %, respectively. Reproducibility of the test was 99.2, and interobserver variation was 8 % with a false positive rate of 3.4 %. The correlation coefficient between the ordinary quantitative assay and the rapid quantitative test was 0.960.

**Conclusions:**

The PSA rapid quantitative test system provided results quickly and was easy to use, so that tests using this system can be easily performed at outpatient clinics or elsewhere. This system may also be useful for initial cancer screening and for point-of-care testing, because results can be obtained within 12 min and at a cost lower than that of conventional quantitative assays.

## Background

Measuring prostate specific antigen (PSA) levels is widely used to identify men with an increased risk of prostate cancer. The serum- or plasma-based immunoassays currently available are associated with time consuming sample processing and the need for sophisticated technical equipment. Therefore, various strip tests for the qualitative and semi-quantitative determination of PSA based on immunochromatographic measurements of serum [1–7] or whole blood [8] have been developed.

Visual assessment of the currently available whole blood assays allows for a yes or no decision without definite information regarding the concentration of PSA in the blood. Although this kind of qualitative assay is sufficient for clinical decision making, prognostic information inherent to the concentration of circulating PSA is lost. Furthermore, inter-individual variability of visual assessment of the test strip at the detection limit of the assay may cause lead to substantial analytical errors [9].

In this study, we evaluated an improved assay for PSA and a newly developed reader to overcome the limitations of previous tests and to enable reliable quantitative and rapid testing for PSA. This tool may be useful for initial cancer screening and may be applied in point-of-care testing.

## Methods

### Patients and serum samples

From June 2014 to May 2015, 305 male patients (mean age 67 years, range 40–98 years), with or without prostate disease were analysed. All of the men were evaluated at E-Da Hospital in Taiwan.

### Control method

For comparative purposes, a blood sample from each patient was collected immediately before the test for use in the standard laboratory method, a chemiluminescent microparticle immunoassay (CMIA), to determine the concentration of PSA. The blood samples were allowed to clot for 1 h at room temperature before being centrifuged. The sera were then immediately analysed using an ABBOTT ARCHITECT *i* System analyzer PSA assay according to the manufacturer’s instructions. The study protocol was approved by the Human Research Ethics Committee of the E-Da hospital, and written informed consent was obtained from each participant before enrolment.

### Principles of the PSA rapid quantitative test system

The PSA rapid quantitative test system includes a special cassette (C.J. Biotec Corp. Pingtung, Taiwan) and a Chromogenic Test Reader (KAIWOOD Technology Co. Ltd., Tainan, Taiwan). The special cassette consists of two different regions: the sample well and the test area. The PSA test strip included the sample pad, conjugated pad, nitrocellulose membrane, absorption pad, and a backing card. The anti-PSA antibody and goat anti-mouse immunoglobulin G antibody defined as the test line and control line were immobilized on the nitrocellulose membrane. The anti-PSA antibody-colloidal gold conjugates were immobilized on the conjugated pad, which was defined as the mixture area. After the sample solution including the PSA antigen in the serum was dropped into the sample pad, the PSA antigen first bonded with the anti-PSA antibody-colloidal gold conjugates and then bonded with the anti-PSA antibody. When the PSA concentration was increased, larger volumes of the colloid gold were aggregated in the test line and this deepens the color of the test line. In addition, excess labeled antibody conjugate will be captured at control line and a second red colored line was also observed of the membrane, indicating the proper test performance. The colored band must be visualized on the control line, so the test could be considered as invalid if there is no color line present in the control region.

**Fig. 1:**
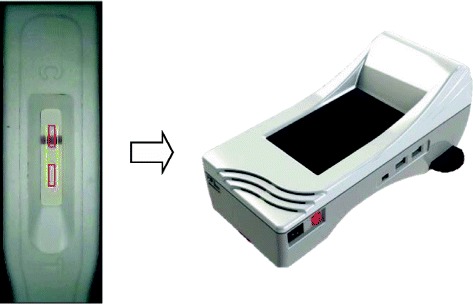
The control line and test line of the prostate specific antigen (PSA) strip were captured by the Chromogenic Rapid Test Reader

**Fig. 2:**
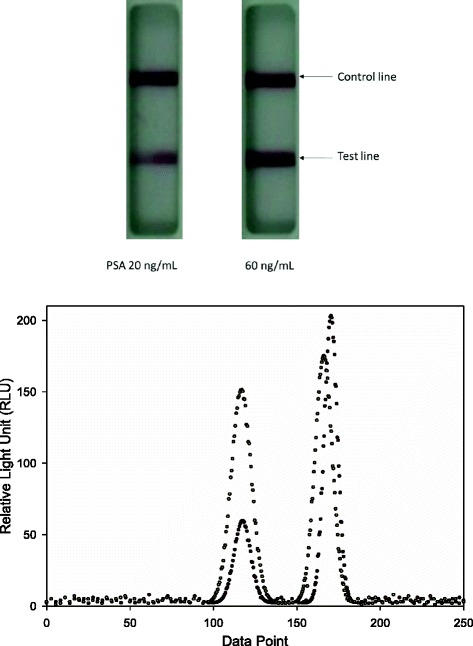
The image of gold immunochromatographic assay (GICA) strip and the curve of the GICA strip signal

The Chromogenic Test Reader mainly consists of an Advanced RISC Machine (ARM) processors, a complementary metal-oxide semiconductor (CMOS) sensor carrier, a set of LED lights, and a test strip carrier. The control and test lines of the PSA strip are captured by the Chromogenic Test Reader (Fig. 1). The test and control lines in the detection zone are recognized by an evaluation algorithm. The intensity of the test line, determined by measuring its reflectance, is directly proportional to the concentration of PSA. The top image in Fig. 2 shows the gold immunochromatographic assay (GICA) strip with PSA concentrations of 20 ng/mL and 60 ng/mL. The bottom image shows the corresponding curve of the GICA strip signal corresponding. The left peak of the strip is the test line, and the other is the control lines. The figure shows that the higher the PSA concentration the higher the peak of the test line. The acquired digital signal after pre-filtering was then processed by the ARM processors. In order to quantitatively analyse the GICA strip, the curve of the strip signal was first segment using the fuzzy C-means algorithm [10, 11] to obtain the location of the test line and control line. In addition, to establish a standard calibration curve for the Chromogenic Test Reader, we used serum samples with various PSA concentrations (60, 30, 20, 15, 10, 7.5, 3.25, 1 ng/mL) were injected into the PSA test strip. The serum samples were prepared by the high PSA concentration (60 ng/mL) of the antigen. The color intensity of test line analyzed by Chromogenic Test Reader was converted via the standard calibration curve and interpolation method into a PSA concentration.

### Test procedure

The PSA test cassette is provided with droppers and a buffer solution. The patient’s fingertip should be swabbed with alcohol and then allowed to dry for 30 s. The fingertip is then pricked with a sterile lancet to obtain one drop of blood, and the droppers are used to draw the blood sample and then transfer it to the sample well on the PSA cassette. After 2 min, two drops of a buffer solution are added to the sample well to allow the blood to migrate. If visible migration across the test area does not start, an additional drop of buffer solution can be added to the sample well. When PSA concentration levels are ≥ 4 ng/mL, a burgundy-coloured band should develop on the test line. For quantitative analysis and to reduce errors, the Chromogenic Test Reader is used to measure the colour signals of the test line, as shown in Fig. 1. Interpretation of the test must be done after 10 min.

### Evaluation procedure

Two urologists who were unaware of the measured PSA values interpreted the tests independently from one another to avoid bias. The observers were also unaware that the two different tests were being performed on the same patients to avoid bias. Positive and negative results of each test were classified as true positive or negative and false negative or positive by comparing them with the quantitative PSA results as measured by the ABBOTT ARCHITECT *i* System analyzer PSA assay, using the threshold value of 4 ng/ml.

### Statistical analysis

All statistical analyses were performed using the Statistical Package for Social Science (SPSS for Windows, version 10.0; SPSS Inc., Chicago, Ill). Sensitivity, specificity, accuracy, positive and negative predictive values of the quantitative PSA test was calculated. Sensitivity was defined as the ratio between the true positive results and the sum of the true positive and false negative results. Specificity was defined as the ratio between the true negative results and the sum of the true negative and false positive results. The positive predictive value was defined as the ratio between the true positive results and the sum of all positive results, and the negative predictive value was defined as the ratio between the true negative results and the sum of all negative results. Accuracy was defined as the ratio between the sum of the true observations (positive and negative) and the total observations. Relationships between GICA method and CMIA method were examined using Pearson’s correlation analyses.

## Results

The patients were divided into two groups on the basis of the conventional quantitative PSA value. Of the 305 patients, 229 (75.1 %) had a PSA value of < 4 ng/mL (median 0.79 ng/mL; range 0.05-3.98 ng/mL) and 76 (25 %) had a PSA value of > 4 ng/mL (median 7.59 ng/mL; range 4.01-169.10 ng/mL). When the patients were divided into two groups on the basis of the rapid quantitative PSA value, of the 305 patients, 221 (72.5) had a PSA value of < 4 ng/mL (median 1.03 ng/mL; range 0.40-4.07 ng/mL) and 84 (27.5 %) had a PSA value of > 4 ng/mL (median 6.33 ng/mL; range 4.10-112.11 ng/mL).

The within-run imprecision experiments (*n* = 9) with the PSA rapid assay yielded CVs between 4.4 and 13.1 % for PSA concentrations between 1.12 and 12.39 ng/mL. The CV for day-to-day imprecision, performed as 9 repetitive measurements on 9 subsequent days, was between 6.6 and 7.3 % (Table 1).

**Table 1:** Analytical performance of the PSA test strip reader

	PSA concentration ng/mL	CV (%)	Sample material
Within-run imprecision (*n* = 9)	1.12	4.4	Blood
	1.20	8.6	Blood
	4.32	4.5	Blood
	4.16	4.4	Blood
	12.39	13.1	Blood
	3	8.3	Control
	5	8.9	Control
Day-to-day imprecision (*n* = 9)	3	6.6	Control
	5	7.3	Control

**Fig. 3:**
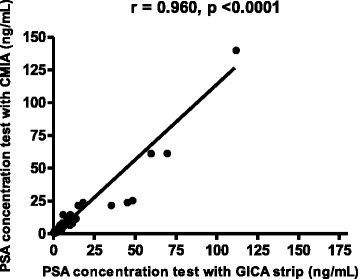
Comparison of prostate specific antigen (PSA) concentrations (ng/mL) obtained with the gold immunochromatographic assay (GICA) and quantitative standard laboratory method (chemiluminescent microparticle immunoassay, CMIA)

The positive and negative results obtained by the two methods are shown in Table 2. In the table, (tp) indicates that both methods classified the test sample as positive; (tn) indicates that both methods classified the test sample as negative; (fn) indicates that the CMIA method classified the test sample as positive but the GICA method classified the test sample as negative; and (fp) indicates that the CMIA method classified the test sample as negative but the GICA method classified the test sample as positive. Sensitivity, specificity, positive and negative predictive values, and accuracy of the PSA rapid quantitative test system were 100 %, 96.6 %, 89.5 %, 100 %, and 97.4 %, respectively. The GICA method had a false positive rate of 3.4 and a false negative rate of 0 % compared to the CMIA method. There was a high level of reproducibility of the GICA test (99.2 %), with an overall concordance rate between the observers of 98 %. The correlation coefficient between the two methods was 0.960 (Fig. 3). In addition, the results of the PSA rapid quantitative test are comparable with those of other PSA one-step tests described in the literature, using serum or capillary blood (Table 3).

**Table 2:** Positive and negative results obtained by the two methods

Results of the GICA method	Results of the CMIA method (gold standard)		
	Positive	Negative	Total
Positive	68 (*tp*)	8 (*fp*)	76
Negative	0 (*fn*)	229 (*tn*)	229
Total	68	237	305

*GICA* gold immunochromatographic assay, *CMIA* chemiluminescent microparticle immunoassay

**Table 3:** Performance of various PSA one-step tests reported in the literature

Author	PSA test	Sample	Time	Sensitivity	Specificity
Dok An et al [1]	One Step PSA^TM^	Serum	15 min	100	90
Jung et al [3]	Chembio	Serum	10 min	67	87
Jung et al. [3]	Medpro	Serum	10 min	87	88
Jung et al. [3]	Syntron	Serum	10 min	93	93
Jung et al. [3]	Seratec	Serum	10 min	80	97
Lein et al. [4]	Tandem-E	Serum	12 min	63	92
Lein et al. [4]	lMx	Serum	12 min	68	95
Lein et al. [4]	LIA-mat	Serum	12 min	83	87
Madersbacher et al. [6]	Oncoscreen®	Serum	10 min	93	93
Berg et al. [8]	One-Step PSA	Serum	10 min	90.5	83.8
Fernández-Sánchez et al. [24]	CanAg	Serum	20 min	87	79
Fernández-Sánchez et al. [24]	Immulite	Serum	20 min	77	83
Berg et al. [8]	Urale®	Capillary whole blood	12 min	91	81
Miano et al. [23]	One-Step PSA	Capillary whole blood	20 min	97.6	90.4
Wu et al.	Quantitative	Capillary whole blood	12 min	100	96.6

## Discussion

In this study, we introduce a rapid quantitative determination method to measure the concentration of PSA using a GICA strip based on the Chromogenic Rapid Test Reader. Comparing the GICA method and the CMIA method revealed a strong correlation coefficient of 0.960 between these two methods. The results suggest that the GICA method may be applicable to quantitatively determine PSA concentrations.

The incidence rate of prostate cancer has increased remarkably. Since radical therapeutic methods are still limited, the early detection and treatment of prostate cancer is essential. Although screening for prostate cancer is still one of the most controversial issue in oncology, it is well recognized that a combination of a digital rectal examination or ultrasonography and a PSA test is the most useful and effective method to diagnose prostatic carcinoma [12–15].

Previously, the widespread use of PSA testing has been reported to be one of the factors that have led to a significant increase in the diagnosis of organ-confined tumours and a decline in prostate cancer mortality rates [16–19]. Furthermore, evaluation of serum PSA level at 45 years of salvage radiotherapy for biochemical relapses after prostatectomy may serve as a significant prognosticator for both biochemical and clinical disease-free outcomes [20]. McLeod DG suggested that PSA testing remains the most efficacious marker available, both to evaluate therapy and to use as a screening tool [21]. In the present study, we introduce an optical inspection system to measure PSA concentrations and a new detection model covering from qualitative to quantitative analysis. Of note, this system is very easy to use and it only needs an initial and brief training to interpret the test results correctly. In addition, it would be useful in the setting of both general practitioner and office urologist. Furthermore, it uses whole blood from a finger prick (30 μl) which is more patient-friendly than traditional venepuncture. In addition, other rapid PSA tests have been reported to require serum samples or capillary whole blood (approximately 80 μl) [1, 6, 22]. Using finger prick whole blood rather than a serum sample saves time (a standard laboratory process to obtain serum needs 1 h to allow for blood clotting after collection, and 10–15 min to separate serum from blood by centrifugation) and produces the final screening results in about 12 min. Another benefit of the PSA rapid quantitative test system is that the cost is significantly lower compared to a conventional quantitative serum PSA test in Taiwan, (about New Taiwan (NT) $300 vs. NT$400). Considering the number of people that could be involved in a screening programme for prostate cancer, the savings could be. Given the intended use of this test (initial prostate cancer screening programme), sensitivity is more important than specificity. The PSA rapid quantitative test system had a very high sensitivity (100 %), even near the cut-off value of PSA of 4 ng/ml. While the overall specificity was 96.6 %, the false-positive rate was 3.4 % with a PSA concentration in the range of 3–4 ng/ml. The accuracy was also very high (97.4 %). The results of the PSA rapid quantitative test are comparable with those of other PSA one-step tests described in the literature [1, 3, 4, 6, 8, 23,24], using serum or capillary blood (Table 3). Another advantage of the PSA rapid quantitative test is that the reader can be linked and send results directly to a hospital’s or healthcare centre’s data management system. As a result, it is now possible to measure highly specific and sensitive results of PSA concentration within 12 min without the need for a sophisticated clinical chemistry environment.

A previous study suggested that a semi-quantitative immunochromatographic test was difficult to perform and that interpretation was difficult. Furthermore, the rate of false strip test results was disappointing even for PSA values far from the cut-off value [25]. Various reasons, apart from the possible technical inferiority of the investigated product, may explain this finding. First, the colour stability of the test is particularly affected by variations in the reading time [3]. Second, the testing of whole blood instead of serum may be associated with less advantageous immunochromatographic properties. Third, well-known differences in the PSA methods [5, 26, 27] used for comparisons may influence the results such as: (1) Antibodies; affinity and specificity for various epitopes of PSA forms [28–30] or cross-reactivity to PSA homologous antigens [31]. (2) Calibration of the assay method. (3) Procedure of the assay; incubation time, equilibrium or kinetics [28, 32], adjuvants (e.g. stabilizing standard preparations, particularly albumin), “high-dose hook” effect. (4) Lot-to-Lot variations in assays [33]. (5) Interference; auto-anti-PSA- antibodies. Fourth, handling and interpretation of the strip tests was mostly performed by trained investigators in other studies. Ultimately, the performance of the test is highly dependent on the study population, particularly with regards to the distribution of PSA values and the prevalence and extent of PSA concentrations exceeding the cut-off value [25]. In the present study, the PSA rapid quantitative test had a higher sensitivity and specificity compared with previous studies [1, 3, 4, 6, 8, 22–24], which may be because the results were interpreted using technical signal detectors and a user with experience in handling and interpreting such detectors, which may overcome the problem that strip test are frequently difficult to read, since the colour reaction in the test field can be weak.

The main limitation of this test is that eight samples with values of less than 4 ng/mL showed false positive results in the range 3–4 ng/ml where the possibility of prostate cancer detection is still about 25 % [34]. The most important and most concerning cause of an elevated PSA is prostate cancer. However, prostate cancer is only one of many potential causes of an elevated PSA. Virtually anything that irritates the prostate will cause the PSA to rise, at least temporarily. The most common cause of PSA elevation includes benign prostatic hyperplasia (enlargement of the prostate, secondary to a noncancerous proliferation of prostate gland cells) and prostatitis (inflammation of the prostate). In fact, PSA elevation can also occur with prostate manipulation such as ejaculation, prostate examination, urinary retention or catheter placement, and prostate biopsy. Hence, elevated the specificity for this PSA rapid quantitative test system, ranging between 2 and 4 ng/mL, is need. Moreover, previous study indicated that the use of free/total PSA ratio in patients with PSA levels of 4–10 ng/mL should enhance the specificity of PSA screening and decrease the number of unnecessary biopsies [35]. However, a free/total ratio is not available with this method. We will test more samples in the near future to further examine the suitability of such kits for mass screening.

## Conclusions

The results of this study showed that this PSA rapid quantitative test with a GICA strip based on a Chromogenic Rapid Test Reader yielded good results and was appropriate for the quantitative determination of PSA concentration. We do not believe that a PSA rapid quantitative test and clinical chemistry tests are mutually exclusive methods. Clearly laboratory-based methods have a better analytical performance [36]. Thus, if time is not critical and the appropriate equipment is available, laboratory-based methods may still be preferable. However, for many clinical situations and for many point-of-care measurements, PSA rapid quantitative testing may be the preferred method, particularly when the results can be sent directly to a hospital data management system as is possible with the PSA Chromogenic Reader.

## Abbreviations

PSA: Prostate specific antigen
CMIA: Chemiluminescent microparticle immunoassay
ARM: Advanced RISC machine
GICA: Gold immunochromatographic assay
